# The human brain in a high altitude natural environment: A review

**DOI:** 10.3389/fnhum.2022.915995

**Published:** 2022-09-15

**Authors:** Xinjuan Zhang, Jiaxing Zhang

**Affiliations:** ^1^Institute of Brain Diseases and Cognition, School of Medicine, Xiamen University, Xiamen, China; ^2^Department of Physiology, School of Medicine, Xiamen University, Xiamen, China

**Keywords:** brain, high altitude, hypoxia, visual cortex, motor cortex, insular cortex, MRI

## Abstract

With the advancement of *in vivo* magnetic resonance imaging (MRI) technique, more detailed information about the human brain at high altitude (HA) has been revealed. The present review aimed to draw a conclusion regarding changes in the human brain in both unacclimatized and acclimatized states in a natural HA environment. Using multiple advanced analysis methods that based on MRI as well as electroencephalography, the modulations of brain gray and white matter morphology and the electrophysiological mechanisms underlying processing of cognitive activity have been explored in certain extent. The visual, motor and insular cortices are brain regions seen to be consistently affected in both HA immigrants and natives. Current findings regarding cortical electrophysiological and blood dynamic signals may be related to cardiovascular and respiratory regulations, and may clarify the mechanisms underlying some behaviors at HA. In general, in the past 10 years, researches on the brain at HA have gone beyond cognitive tests. Due to the sample size is not large enough, the current findings in HA brain are not very reliable, and thus much more researches are needed. Moreover, the histological and genetic bases of brain structures at HA are also needed to be elucidated.

## Introduction

High altitude (HA) is characterized as having lower atmospheric oxygen pressure, a cold climate, and strong ultraviolet rays, and is therefore inhospitable to human settlement. However, there are still a large number of people living in HA regions. More than 10 million highlanders permanently reside above 2,200 m in the Qinghai-Tibet Plateau (Kayser and Wu, 2006). Every year, hundreds of thousands of people move from lowlands to HA regions for work or study staying for several months to several years. Moreover, altitude training has been used as a routine in endurance sports around the world to improve performance (Lundby and Robach, 2016), and thus the effects of exercise under HA hypoxia on the brain may be most important for athletes performing HA training.

**FIGURE 1:**
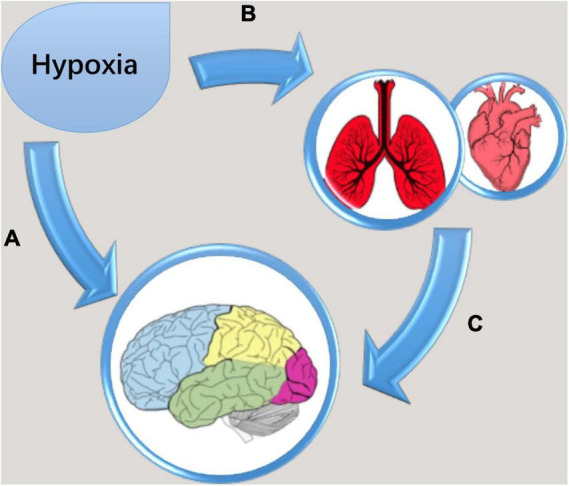
Diagrammatic drawing of cumulative effects of hypoxia on the brain. **(A)** Neurons in the brain directly suffer from the stress of low oxygen concentration; **(B)** cardiovascular and respiratory systems changed in adaptation to hypoxia; **(C)** through afferent feedback, the adaptation in the cardiovascular and respiratory systems act on their control centers in the brain.

The brain accounts for roughly 2% of the total mass of human body, yet it consumes over 20% of the oxygen that the human body intakes (Raichle, 2010), and as a consequence, HA inevitably challenges the brain. Through afferent feedback, the adaptation that occurs in the cardiovascular and respiratory systems (Hoiland et al., 2018) may act on their corresponding control centers in the brain (Figure 1). Moreover, the changes in blood CO_2_ and pH may have direct effect on cerebral blood flow. Hypobaric and cold may be the other two important factors contributing to the alteration of the brain at HA. Aggravation of mental illness (Mizoguchi et al., 2011), impairment of visual sensitivity (Degache et al., 2012), development of acute mountain sickness (AMS) (DiPasquale et al., 2015), and damaged brain structure (Simmons et al., 2015) are more often seen with hypobaric vs. normobaric hypoxia. The cold ambient temperature at HA may result in hypothermia; however, hypothermia has been shown to have a neuroprotective effect against hypoxic encephalopathy (Gao et al., 2014; Kvistad et al., 2014). Most present-day HA natives are descendants of colonizers who arrived in HA regions tens of thousands of years ago. Positive natural selection in the Egl nine homolog 1 (EGLN1), endothelial PAS domain protein 1 (EPAS1), and peroxisome proliferator activated receptor alpha (PPARA) genes, all of which are associated with the hypoxia-inducible transcription factor (HIF) pathway (Simonson et al., 2010; Yi et al., 2010; Yang et al., 2017) has recently been found to be the mechanism underlying the adaptation of Tibetans, who have lived on the Qinghai-Tibet Plateau for millennia. The purpose of the present review was to assess how the combined action of a variety of HA environmental factors and genetic factors lead to changes in various regions of the brain.

Cerebral edema was first associated with HA due to the appearance of many mountain sickness symptoms during HA exposure (Ravenhill, 1913). In an earlier research program named “the American Medical Research Everest Expedition,” finger-tapping behavior was found to be impaired in climbers (West, 1984). After that, along with the advancement of many *in vivo* imaging techniques and analysis methods, more information about changes in brain structure and function in HA populations have been revealed. The present review focused on these new findings relating to the human brain in a natural HA environment. Acute HA exposure typically refers to being on an elevated plateau for several days to weeks, while chronic HA exposure refers to extended living or having permanent residence on a plateau (Javier et al., 2004). Therefore, this review presents a mix of acute exposure, chronic exposure, and life-long exposure literature.

## Brain researches selected and search methodology for this review

Using “high altitude” or “plateau” with “brain” or “cerebral cortex” as keywords limited in Abstract, we retrieved 1,316 items in PubMed and 1,036 items in Web of Science. Finally, a total of 185 articles were distinguished to be related to brain research. These studies involve many aspects of the brain, including brain edema, blood-brain barrier injury, and protective treatments of drugs on brain injury. Brain edema presents extensive whole brain swelling. However, humans would like to know which brain regions were more vulnerable to injury and the cognitive impairments resulting from brain region damage. In fact, HA brain edema has been reviewed by researchers every few years (Hackett and Roach, 2004; Turner et al., 2021), so this review was no longer include the study of brain edema. In addition, there were several animal studies through exposing animals to a simulating HA hypoxia but not real HA environment, which did not include in this review. Magnetic resonance imaging (MRI) and electroencephalography (EEG) are the two advanced technologies to study the brains of living humans currently. MRI has high spatial resolution and can accurately locate millimeter level of brain regions with gray matter (GM), white matter (WM), and cerebrospinal fluid (CSF) can be accurately visualized. Nowadays, functional MRI (fMRI) and EEG have been intensively employed to explore neuronal activities within brains. Therefore, this review mainly summarizes the researches that used these two methods. With these considerations in mind, a total of 53 research articles from PubMed and Web of Science meet the criterion of this review (see Tables 1, 2).

**TABLE 1:** Brain structures in high altitude (HA) people.

References	Location (altitude (m), duration)	Group: number (M/F)	Age (years)	MRI (scanning sequence, magnetic field intensity)	Analysis	Changed structures of the brain regions
Di Paola et al., 2008	Everest or K2 (7500), 15 days	HA: 9 (9/0) world-class mountain climbers	37.9 (31–52)	T2, T1, FLAIR at 1.5 T	Whole brain analysis	Reduced GM volumes in the left angular gyrus (*p* = 0.003) Reduced WM volumes in the left pyramidal tract near the primary and supplementary motor cortex (*p* = 0.002).
		SL:19				
Kottke et al., 2015	Mount Himlung Himal (7126), 23 days	HA: 38 (20/18) climbers longitudinal	45.4 (24–69)	FLAIR, SWI, MDEFT at 3.0 T	Whole brain analysis	Increased cerebrospinal fluid (0.34% [95% CI 0.10–0.58], *p* = 0.006) Reduced WM fraction reduction (−0.18% [95% CI –0.32–−0.04], *p* = 0.012). GM fraction remained stable (−0.16% [95% CI −0.46–0.13], *p* = 0.278).
Zhang et al., 2012	6206, 30 days	HA: 14 (8/6) climbers longitudinal	21 (19–23)	T1, DTI at 1.5 T	Whole brain analysis	No significant regional changes in GM and WM volumes; Decreased FA in the bilateral corticospinal tract, corpus callosum, reticular formation of dorsal midbrain, left superior longitudinal fasciculus, right posterior cingulum bundles, and left middle cerebellar peduncle (*p* < 0.05); Increased RD in the left superior longitudinal fasciculus within the inferior parietal lobule (*p* < 0.05).
Foster et al., 2015	Ev-K2-CNR Pyramid Laboratory (5050), 3 weeks	HA: 7 (5/2) longitudinal	31.8	T1, T2, FLAIR, SWI, CVR at 3.0 T	Whole brain analysis	Decreased whole brain volume (+ 0.4 ± 0.3%, *P* < 0.01); Decreased GM volume (−2.6 ± 1.0%, *P* < 0.01); No changed in WM; Increased CVR in the brainstem (+ 30 ± 12%), hippocampus (+ 12 ± 3%), and thalamus (+ 10 ± 3%)
Verges et al., 2016	Mont Blanc (4350), 6 days	HA: 11 (11/0) longitudinal	28	T1, DWI, ASL at 3.0 T	Whole brain analysis	Not change in GM; Increased WM volume (+ 0.7 ± 0.4%, *p* = 0.005) and diffusion (+ 1.7 ± 1.4%, *p* = 0.002); Reduced cerebrospinal fluid volume (–1.4 ± 1.1%, *p* = 0.009).
Chen X. et al., 2017	Lhasa (3650, Tibet), 0/1/2 years	HA: 69(48/21) longitudinal	18 (17–20)	T1, rs-fMRI at 3.0 T	Whole brain analysis	Decreased GM volume in the left putamen (*p* < 0.05).
Zhang et al., 2010	Qinghai-Tibet Plateau (2616–4200), natives	HA: 28(12/16) SL: 28(12/16)	20.4 (17–22) 20.9 (17–23)	T1, DTI at 3.0 T	Whole brain analysis and ROI	Decreased GM in the bilateral anterior insular cortex, right anterior cingulate cortex, bilateral prefrontal cortex, left precentral cortex, and right lingual cortex (*p* < 0.01); Higher FA in the bilateral anterior limb of internal capsule, bilateral superior and inferior longitudinal fasciculus, corpus callosum, bilateral superior corona radiata, bilateral anterior external capsule, right posterior cingulum, and right corticospinal tract (*p* < 0.05).
Zhang et al., 2013b	Qinghai-Tibet Plateau (2300–4400), 2 years	HA: 16 (16/0)Tibetan SL: 16(16/0)	20 (20–22) 20 (17–22)	T1, DTI at 3.0 T	Whole brain analysis	No changes in total volumes of GM, WM, and cerebrospinal fluid. Decreased GM volumes in the right postcentral gyrus and right superior frontal gyrus, while increased GM in the right middle frontal gyrus, right parahippocampal gyrus, right inferior and middle temporal gyri, bilateral inferior ventral pons, and right cerebellum crus1(*p* < 0.05); Decreased CT in the right superior frontal gyrus (*p* < 0.001).
Zhang et al., 2013a	Qinghai-Tibetan Plateau (2900–4700), Tibetan	HA: 21(6/15) Control: 21(6/15)	16.5 (15–18) 16.3 (15–18)	T1, DTI at 3.0 T	Whole brain analysis	Increased GM volume in the left insula, left inferior parietal gyrus, and right superior parietal gyrus (*p* < 0.001); decreased GM in the left precentral cortex and multiple sites in cerebellar cortex (*p* < 0.001); decreased WM volume was found in the right superior frontal gyrus (*p* < 0.001). Higher FA in a broad range of brain areas (*p* < 0.05). Decreases MD in most WM tracts (*p* < 0.05).
Fan et al., 2016	Dangxiong city (4300, Tibet), 30 days	HA: 31 (16/15) Control: 21	19.7	T1,T2, DTI at 3.0T	Whole brain analysis	Decreased cortical thickness in the bilateral superior frontal gyrus, rostral anterior cingulate gyrus, superior parietal gyrus, supramarginal gyrus, and insula, left fusiform gyrus, and right inferiorparietal gyrus and increased in the bilateral pericalcarine gyrus and precentral gyrus (*p* < 0.05). Increased curvature was significantly in the bilateral precentral gyrus, superior frontal, supramarginal gyrus, inferior frontal gyrus, paracentral lobule, precuneus, superior parietal cortex, temporal gyrus, parahippocampal gyrus, insula, and fusiform gyrus and decreased in the postcentral gyrus and right cingulate gyrus (*p* < 0.05). Increased NBV (*p* < 0.001), GMV (*p* = 0.003), and WMV (*p* < 0.001).
Wei et al., 2017	Qinghai- Tibet Plateau (2300–5300), Tibetan	HA: 77(34/43) Control: 80(34/46)	14–18 14–18	T1,T2 at 3.0 T	Whole brain analysis	Decreased CT in the left posterior cingulate cortex, lingual gyrus, superior parietal cortex, precuneus, rostral middle frontal cortex, right medial orbitofrontal cortex, lateral occipital cortex, precuneus, and paracentral lobule (*p* < 0.05); Decreased curvature in the left superior parietal cortex and right superior marginal gyrus; Increased depth of sulcus in the left inferior temporal gyrus, while decreased depth of sulcus in the right superior marginal gyrus, superior temporal gyrus, and insular cortex (*p* < 0.05).
Chan et al., 2012	3600, a few hours	A men	-	T1, magnetic field intensity unknown	Directly observe	He was confirmed ischaemic infarcts in the medial left occipital lobe and left thalamus.
Fayed et al., 2006	Mt. Everest (8848), Mt. Aconcagua (6959), Mont Blanc (4810), Mt. Kilimanjaro (5895)	HA: 35 climbers SL: 20	33.8 (22–46)	T1,T2, FLAIR at 1.5 T	Directly observe	Only 1 in 13 of the Everest climbers had a normal MRI; the amateur showed frontal subcortical lesions, and the remainder had cortical atrophy and enlargement of Virchow-Robin spaces but no lesions.
Garrido et al., 1993	7000	HA: 26 SL: 21	35 (25–42)	T2 at 1.5 T	Directly observe	Almost half of the climbers showed MRI abnormalities (46%), 5 subjects had cortical atrophy.
Garrido et al., 1995	Over 7500	HA:9(7/2)	34	T1,T2, magnetic field intensity unknown	Directly observe	Five climbers MRI abnormalities (high signal areas, cortical atrophy).
Garrido et al., 1996	Over 8000	HA: 7 natives 21 lowland elite climbers SL:21	33 31	T1,T2 at 1.5 T	Directly observe	Thirteen of lowland climber (61%) and 1 Sherpa (14%) showed MRI abnormalities (mild cortical atrophy, high signal areas in the white matter)
Anooshiravani et al., 1999	Over 6000	HA:8(8/0) climbers	31–48	T1,T2 at 0.5 T	Directly observe	Did not observe the changes in brain imaging.
D’Arrigo et al., 2019	4000, 36 h	A women	50	FLAIR, DWI, magnetic field intensity unknown	Directly observe	Showed abnormal hyperintense signaling in the right insular lobe and smaller similar lesions in the right temporal lobe and in the contralateral insular lobe.
Miyamura et al., 2007	3700	A women	71	T1, magnetic field intensity unknown	Directly observe	Revealed localized lesions at the globus pallidus, bilaterally.
Chen et al., 2019a	Lhasa (3650, Tibet), 2 years	HA: 49(32/17) longitudinal SL:49(32/17)	17–20 17–20	T1,DWI at 3.0 T	Whole brain analysis	FA increased in the regions of right posterior corona radiate, anterior corona radiate, splenium of corpus callosum, decreased in the regions of superior longitudinal fasciculus, right anterior limb of internal capsule, body of corpus callosum (*p* < 0.05).
Chen et al., 2019b	Lhasa (3650, Tibet), 2 years	69	17–20	Rs-fMRI at 3.0 T	Whole brain analysis	The regions with highest contributions to the predictions of psychomotor function were bilateral putamen and bilateral pallidum (*p* < 0.05).
McGuire et al., 2016	U.S. Air Force U-2 pilots(8534–914)	HA:102 (100/2) SL:114 (109/5)	37.9 (28–50) 34.9 (26–50)	T1, FLAIR DTI at 3.0T	Whole brain analysis	Pilots had higher FA values in the fronto-occipital tract where FA values positively correlated with visual-spatial performance scores (*p* < 0.05).
Chen et al., 2016	Qinghai-Tibet Plateau (2300–4400),2 years	HA: 16 SL: 16	20.5 (19–22) 19.9 (19–22)	Whole brain analysis at 3.0 T	DTI	Increased the path length of the commissural fibers connecting homotopic visual areas (*p* < 0.05).
McGuire et al., 2013	high-altitude U-2 pilots	HA:102 SL:91	37 (28–50) 36 (28–50)	T1,T2 at 3.0 T	Whole brain analysis	U-2 pilots demonstrated an increase in volume (394%; *p* = 0.004) and number (295%; *p* < 0.001) of WM hyperintensities.
Guo et al., 2020	Lhasa (3650, Tibet),Tibetan	HA:135(62/73)	19.9 (17–23)	T1, DTI at 3.0 T	Whole brain analysis and ROI	No significant effects were observed for any SNP on any global or intermediate metric from any type of structural and functional network in Tibetan participants (p > 0.05). Positive correlation for the GAS over all 45 HA adaptive SNPs with inter-module communication scores between the right frontal and parietal module and the left occipital module (*r* = 0.283, *p* < 0.05).
Kühn et al., 2019	4554, 12 h and 7 days	HA:10(5/5) longitudinal	41	T1, magnetic field intensity unknown	Whole brain analysis	12 h after descent HA had increased GM and WM (*p* < 0.05). 7 days after descent HA increased cerebral tissue volume changes in the paracentral gyrus region, postcentral gyrus, thalamic area of the left brain; and putamen (*p* < 0.05).
Chen X. et al., 2017	Qinghai-Tibet Plateau (4200), 4 weeks	29 longitudinal	20 (19–21)	QSM, DTI at 3.0 T	ROI	Increased iron concentration in basal ganglia, including caudate nucleus, putamen, globus pallidus and substantia nigra; FA increased and the MD decreased after HA exposure in caudate nuclei, putamen, globus pallidus, substantia nigra, red nucleus, dentate nucleus, with linear dependence on iron concentration only in putamen.
Yan et al., 2010	Qinghai-Tibet Plateau (2616–4200), 20 years	HA: 28(12/16) SL:28(12/16)	20 (17–24) 20 (18–24)	T1,DTI at 3.0 T	Whole brain analysis and ROI	Decreased GM volume at bilateral anterior insula, bilateral prefrontal cortex, the left precentral, the left cingulate and the right lingual cortex (*p* < 0.01). Decrease of the FA value in the right posterior cingulum and the right precentral cortex (*p* < 0.01).

M, male; F, female; CT, cortical thickness; DWI, diffusion-weighted imaging; DTI, diffusion tensor imaging; FA, fractional anisotropy; FDR, false discovery rate; FLAIR, fluid attenuated inversion recovery; FWE, family wise error; GM, gray matter; MD, mean diffusion; MRI, magnetic resonance imaging; QSM, quantitative susceptibility mapping; RD, radial diffusivity; TBSS, tract-based spatial statistics; VBM, voxel-based morphometry; WM, white matter; T, tesla.

**TABLE 2:** Brain functions in high altitude (HA) people.

References	Location (altitude (m), duration)	Group: number (M/F)	Age (years)	Analysis	Techniques/tasks	Changed functions of the brain regions
Rostrup et al., 2005	Chacaltaya Mountain (5260), 5 weeks	HA:11(6/5) longitudinal	26.3 (22.4–34.4)	Whole brain analysis and ROI	visual task fMRI at 1.5 T machine/white rectangular checkerboard	Reduced 23% magnitude of the BOLD signal in visual task (*p* = 0.02). Decreased coefficient ADC (*p* < 0.05).
Yan et al., 2011b	Qinghai-Tibet Plateau (2527–3985), natives	HA: 12 SL: 11	22.4(20–24) 24.8(22–27)	Whole brain analysis	fMRI at 3.0 T machine/inspiration task	Lower CVR in the primary motor, visual cortex, somatosensory cortex, precuneus, posterior cingulate cortex, thalamus, and caudate; longer delayed hemodynamic response (*p* < 0.05).
Yan et al., 2011c	Qinghai-Tibet Plateau (2616–4200), natives	HA: 10 SL: 9	–	Whole brain analysis	visual task fMRI at 3.0 T machine/Food picture	Decreased BOLD signals in the middle, superior, and inferior frontal gyrus, insular cortex, cuneus, middle occipital gyrus, lingual gyrus, cingulate gyrus, and precuneus (*p* < 0.05); Increased BOLD signals in the inferior parietal lobule and cerebellar tonsil (*p* < 0.05).
Yan et al., 2011d	Qinghai-Tibet Plateau (2616–4200), natives	HA: 28(12/16) SL: 28(12/16)	20(19–22) 21(19–22)	Whole brain analysis and ROI	visual task fMRI at 3.0 T machine/2-Back verbal memory	Decreased BOLD signals in the inferior and middle frontal gyrus, middle occipital gyrus, lingual, the pyramis of vermis, and thalamus (*p* < 0.05).
Yan et al., 2011a	Qinghai-Tibet Plateau (2616–4200), natives	HA: 28(12/16) SL: 28(12/16)	20(19–22) 21(19–22)	Whole brain analysis and ROI	visual task fMRI at 3.0 T machine/2-Back spatial memory	Increased BOLD signals in the left pyramis, left superior temporal gyrus; Decreased BOLD signals in the left middle occipital gyrus (*p* < 0.05).
Wang et al., 2014	Lhasa (3650, Tibet), 3 years	HA: 18(8/10) SL: 18(9/9)	22(20–24) 23(21–24)	ROI	visual task ERP/visual voluntary attention task	Found bilateral N1 activity; Smaller P3 under high perceptual load (*p* < 0.05).
Ma et al., 2015b	Lhasa (3650, Tibet), 2 years	HA:20(10/10) SL: 20(10/10)	21.8(21–24) 22.8(21–24)	ROI	visual task ERP/Go no go	Larger N2amplitude and smaller P3 amplitude in both the go and no go condition (*p* < 0.001).
Ma et al., 2015a	Lhasa (3650, Tibet), 2 years	HA: 17(8/11) SL: 17(9/8)	20.75 (21–24) 21.78 (21–24)	ROI	visual task ERP/Go no go	Larger ERN(*p* = 0.027) and CRN (*p* = 0.0001).
Ma et al., 2015c	Lhasa (3650, Tibet), 2 years	HA: 21(9/13) SL: 21(10/11)	21.95 (19–24) 21.9(19–24)	ROI	visual task ERP/Flanker	Smaller P3 amplitude in the incongruent target (*p* < 0.05).
Zhao et al., 2016	Lhasa (3650, Tibet), 30 days	HA:25(25/0) longitudinal	24.6 (21–28)	ROI	EEG	Decreased theta power after ascending to HA for 7 days (*p* < 0.05); Increased alpha and beta powers but decreased delta power in the middle posterior parietal and occipital areas after staying at HA for 30 days (*p* < 0.05); Decreased alpha power and increased beta power after return to sea level for 7 days (*p* < 0.05).
Chen et al., 2016a	Qinghai-Tibet Plateau (2300–4400),2 years	HA: 16 SL: 16	20.5(19–22) 19.9(19–22)	Whole brain analysis	fMRI at 3.0 T machine/ Resting-state	Increased Reho in the right inferolateral sensorimotor cortex (*p* < 0.05).
Zhang et al., 2017	Qinghai-Tibet Plateau (2300–4400), 2 years	HA: 16 SL: 16	20.5(19–22) 19.9(19–22)	Whole brain analysis	fMRI at 3.0 T machine/ Resting-state	Increased ALFF in the bilateral occipital cortex, lingual gyrus, cuneus, and fusiform gyrus; Decreased ALFF in the right anterior insular cortex, extending to the caudate, putamen, inferior frontal orbital cortex, temporal pole, and superior temporal gyrus (*p* < 0.05).
Chen X. et al., 2017	Lhasa (3650, Tibet), 0/1/2 years	HA: 69(48/21) longitudinal	(17–20)	Whole brain analysis and ROI	fMRI at 3.0 T machine/ Resting-state	Decreased ReHo in the superior temporal gyrus, superior parietal lobule, anterior cingulate gyrus, and medial frontal gyrus; Increased ReHo in the hippocampus (*p* < 0.01).
Chen et al., 2016b	Qinghai-Tibet Plateau (2300–4400), 2 years	HA: 16 SL: 16	20.5(19–22) 19.9(19–22)	Whole brain analysis	fMRI at 3.0 T machine/ Resting-state	Increased VMHC in the bilateral visual cortex (*p* < 0.05).
Ma et al., 2018a	Lhasa (3650, Tibet), 2 years	HA: 29 SL: 29	(18–25)	ROI	visual task ERP/Visual voluntary attention	Slower response time in stimulus–driven attention, smaller P1/N1/P3 amplitude (*p* < 0.05).
Ma et al., 2018b	Lhasa (3650, Tibet), 2 years	HA:32 (15/17) SL:33(17/16)	(19–22)	ROI	visual task ERP/Mental rotation	Slower response time in the mental rotation effect, larger rotation related negativity amplitude in each rotation angle condition (*p* < 0.05).
Zhang et al., 2018a	Lhasa (3650, Tibet), 2 years	HA: 20(10/10) SL:22(11/11)	22.7(21–25) 23.0(23–25)	ROI	visual task ERP/Visual search	Longer reaction time, lower N2pc amplitude, larger N2cc amplitude, higher MP amplitudes, and longer RAP latency (*p* < 0.05).
Zhang et al., 2018b	Qinghai-Tibet Plateau (2900–4200), natives	4200m:23 (12/11) 3700m:21 (11/10) 2900m:23 (12/11)	21(18–23) 21(18–23) 21(18–23)	ROI	visual task ERP/Attention network	Decreased orienting and overactive executive functions in 4200m residents (*p* < 0.05).
Richardson et al., 2011	La Paz (3700), natives	HA: 22(11/11) SL: 18(7/11)	13–16	ROI	EEG	Showed reductions in delta and beta frequency amplitudes (*p* < 0.05).
Ma et al., 2019b	Lhasa (3650, Tibet), 2 years	HA: 19(10/9) SL: 21(9/13)	22.3 22.1(20–24)	ROI	visual task ERP/2-Back verbal and spatial memory	Decreased late-positive potential amplitude in verbal and spatial tasks and larger P2 in spatial memory (*p* < 0.05).
Ma et al., 2019a	Lhasa (3650, Tibet), 2 years	4500m:17(9/8) 3700m:18(8/10) 2700m:17(10/7)	21.7 (21–24) 20.64 (18–24)	ROI	visual task ERP/flanker	The N2 difference wave was smaller in the 4500m group than in the groups living below 4000 m (*p* < 0.05).
Wang et al., 2017	Qinghai-Tibet Plateau (2200), 1 years	19(9/10) longitudinal	24.8	Whole brain analysis and ROI	fMRI at 3.0 T machine/Resting-state	ALFF increased in Left middle frontal gyrus, decreased in the right lingual gyrus; ReHo increased in left superior frontal gyrus, decreased in right precuneus and parietal lobe (*p* < 0.05).
Li et al., 2021	Lhasa (3650, Tibet), 2 years	HA:30(13/17) SL:30(16/14)	(20–25) (17–22)	ROI	visual task ERP/mental rotation	Decreased P50 mean amplitude and the CDA amplitude of mental rotation mainly in the occipital, parietal and frontoparietal. Increased the beta/alpha power in mental rotation and the theta/alpha/beta power in CDA;
Xiang et al., 2021	Lhasa (3650, Tibet), 2 years	HA:32(15/17) SL:32(17/16)	(19–22)	ROI	visual task ERP/mental rotation	Decreased the alpha and the beta ERD within the time window (400–700 ms) (*p* < 0.01). The decreased ERD was observed at the parietal–occipital regions within the alpha band and at the central–parietal regions within the beta band (*p* < 0.01).
Xin et al., 2020	Lhasa (3650, Tibet), 2 years	HA:69(48/21) longitudinal	18.2(17–19)	Whole brain analysis	fMRI at 3.0 T machine/Resting-state	Decreased degree centrality and nodal efficiency in the insula, cingulum, hippocampus, amygdala, putamen, thalamus, temporal, and vermis; Increased degree centrality and nodal efficiency in the frontal, occipital, parietal, and angular (*p* < 0.05).
Chen et al., 2021	Lhasa (3650, Tibet), 1 and 2 years	HA:69(48/21) longitudinal	18.2(17–19)	Whole brain analysis	fMRI at 3.0 T machine/Resting-state and stroop	Decreased degree of co-activation within the left/right frontoparietal network, sensorimotor network, and auditory network after exposure. Which was found in left angular gyrus, in the right frontoparietal network, in left precentral gyrus and postcentral gyrus, in left middle frontal gyrus and left superior temporal gyrus (*p* < 0.001).
Forster et al., 1975	4300 (Mt. Evans, colo), 12 days	7(7/0)	-	ROI	EEG/visual evoked responses	In three subjects, EEG frequency was increased, amplitude decreased. In 4 subjects, visual evoked responses decreased.
Qiu et al., 2021	Dangxiong (4300), 1 month	23(12/11) longitudinal	19.4(19–21)	ROI	visual task ERP/clock	Decreased N1 and P3 at occipital electrodes. (*p* < 0.05) Decreased theta power. (*p* < 0.05)
Nisha et al., 2020	6096, mean 10.5 h/annum	HA:4(3/1) SL:4(3/1)	37.23	Whole brain analysis	visual task fMRI at 3.0 T machine/working memory	Showed significant activation in the right middle frontal gyrus (*p* < 0.05).

ALFF, amplitude of low-frequency fluctuations; BOLD, blood oxygenation level dependent; CDA, contralateral delay activity; CRN, correct-related negative; CVR, cerebral vascular reactivity; ERN, error-related negative; ERD, event-related desynchronization; ERP, event-related potential; HA, high altitude; MRI, magnetic resonance imaging; Reho, regional homogeneity; T, tesla.

## Brain structures at high altitude

### Gray matter and white matter changes

In a lot of earlier studies, high intensity signals on T2-weighted MR images were used to directly observe the severe damage of brain GM and WM. Cytotoxic edema, as in early hypoxic encephalopathy, leads to restriction of water motion, producing high signal intensity on T2-weighted MR images. Subcortical edema and cortical atrophy (enlarged cerebral sulci) were reported in groups of 26 and 21 mountain climbers ascending to altitudes of 7,000 and 8,000 m (Garrido et al., 1993, 1996) and in 35 climbers after ascents to peaks of different heights ranging from Mont Blanc (4,810 m) to Mt. Everest (8,848 m) (Fayed et al., 2006) compared with non-climbers. In a comparative longitudinal study, high-intensity signal and cortical atrophy were also detected in the occipital cortex in 2 of 9 climbers after reaching altitudes between 7,800 and 8,463 m (Garrido et al., 1995). However, no severe damage visible to the naked eye in brain imaging was observed in eight male climbers between 31 and 48 years of age a few days before and between 5 and 10 days after returning to sea level following ascent to altitudes of over 5,947 m (Anooshiravani et al., 1999), which may be due to small number of climbers and low intensity magnet which was a 0.5 T. Brain region identified by direct observation may be the brain region most affected by hypoxia. In general, serious brain structural damage can be found through direct observation, but the changes that are invisible to the naked eye need more detailed analysis.

In recent years, GM and WM structural changes in individuals at HA have been examined based on brain T1 MRI and diffusion tensor imaging (DTI), respectively (detailed information is shown in Table 1). GM volume, cortical thickness, and cortical surface area were quantitatively analyzed using voxel-based morphometry (VBM), while WM microstructure was measured using Tract-Based Spatial Statistics (TBSS). TBSS measures the fractional anisotropy (FA) and mean diffusion (MD) of WM fibers. FA represents the ratio between the length of the primary axis and the other two orthogonal axes, with a high anisotropy representing diffusion that is highly oriented in one direction. MD represents the overall free space available for water to self-diffuse, and thus is the average length of all three axes.

There were no consistent results regarding the alteration of the total brain volume. Total brain volume was first found unchanged after mountain climbers stayed at the Tanggula Mountains (6,260 m) for 30 days (Zhang et al., 2012) and at Mount Himlung Himal (7,126 m) for 23 days (Kottke et al., 2015). More recently, total brain volume was revealed to be reduced after HA exposure for 3 weeks at 5,050 m (Foster et al., 2015). Global GM volume, however, was found to increase after 30 days at 4,300 m (Fan et al., 2016). High altitude climbers suffers from cortical atrophy (Fayed et al., 2010). This inconsistency may be due to the variability of study designs. In studies conducted by Zhang et al. (2012) and Kottke et al. (2015), brain images were obtained before the start of the expedition and then several weeks after the return to sea level. Thus, the brain structure could have been influenced by reoxygenation-induced changes in cerebral blood flow (CBF) (Harris et al., 2013; Wang et al., 2018). In contrast, Fan et al. (2016) conducted the study at both the lowland and the plateau.

Regional GM changes were seen in the mountain climbers who repeated expeditions to Mount Everest (Di Paola et al., 2008), adult soldiers who had garrisoned the frontiers in the Qinghai-Tibet Plateau for 2 years (Zhang et al., 2013b), climbers who made a single ascent to for less than 36 h (D’Arrigo et al., 2019), and college freshmen who immigrated to the Qinghai-Tibet Plateau and were followed-up for 2 years (Chen X. et al., 2017, Chen et al., 2019a). To explore the influences of the environment on HA residents, Han immigrant adolescents (Zhang et al., 2010) and HA adolescents (Yan et al., 2010; Wei et al., 2017) living in the Qinghai-Tibet Plateau were studied, and decreased GM volume and changes in cortical thickness were detected. Two follow-up observations have also been conducted to investigate the persistent sequelae to brain structure after people return to lowlands. An earlier observation is from the study on college students who volunteered for a 30-day teaching on the Qinghai-Tibet Plateau (Fan et al., 2016), and a latter observation examined volunteers 12 h post-descent and again 3.5 months after a 7-day HA exposure at 4,554 m, respectively (Kühn et al., 2019). Among these HA-exposed populations, GM volume and cortical thickness showed discrepant changes, with some regions having increased but others having decreased.

For HA immigrants, the increase in GM may be associated with hypoxia-induced neurogenesis, gliogenesis, or vascular proliferation. Adult neocortices, such as the prefrontal cortex, inferior temporal cortex, and posterior parietal cortex, have the capability of neurogenesis (Gould et al., 1999), which may potentially be induced by stress (Magavi et al., 2000) or afferent feedback (function-activated effects). The brain is the source of behavior, but in turn, it is modified by the behaviors it produces. Glial cells comprise more than 85% of the total number of brain cells. They are sensitive to changes in oxygen partial pressure (Angelova et al., 2015), and can be activated by hypoxia (Pforte et al., 2005). Vasculature accounts for about 5% of GM (Zatorre et al., 2012). The capillary length per unit volume of tissue, dilation, and density in the cortex increased after 3 weeks of exposure to a hypoxic environment (LaManna et al., 1992; Boero et al., 1999). On the other hand, a decrease in cortical GM may be due to neuronal loss (Maiti et al., 2007). Therefore, unbalanced development between angiogenesis/gliogenesis and neuronal loss could determine regional cortical volume.

Few studies have observed changes in brain WM at HA. The change in total WM volume varies, as seen by increases after people stayed at 4,300 m for either 30 days (Fan et al., 2016) or 6 days (Verges et al., 2016), decreases after ascending Mount Everest (8,848 m) for several days (Di Paola et al., 2008) and Mount Himlung Himal (7,126 m) for 23 days (Kottke et al., 2015), no change after an expedition to 5,050 m for 3 weeks (Foster et al., 2015) and 6,206 m for 1 month (Zhang et al., 2012). The differing results may be due to different experimental conditions. In some studies, brain MRI scans were conducted before individuals ascended to and remained at a plateau after HA exposure (Fan et al., 2016; Verges et al., 2016). In this situation, the vasogenic edema that occurred in WM during HA exposure may have contributed to the increase in WM volume. In other studies, the images were obtained several days after the individuals returned to the lowlands, and thus any brain edema could have already been alleviated (Di Paola et al., 2008; Foster et al., 2015; Kottke et al., 2015). One study showed that total WM volume did not change after an ascent to 6,260 m for 30 days, but regional FA and radial diffusivity values both decreased (Zhang et al., 2012), indicating that only WM microstructure was impaired. Moreover, the number of commissural fibers connecting the bilateral visual cortices increased in adults who had immigrated to and remained on a plateau for 2 years (Chen et al., 2016b). Changes of FA of WM fibers were observed in a lot of studies on descendants of Han immigrants (Zhang et al., 2010), Tang-ku-la Mountains climbers (Zhang et al., 2012), soldiers who have garrisoned the frontiers in Qinghai-Tibet Plateau (Zhang et al., 2013b), subjects as volunteer teachers (Fan et al., 2016), and college freshmen who immigrated to Tibet and followed up for 2 years (Chen et al., 2019a), with some fibers have increased FA but others have decreased FA (Table 1).

The change in cerebrospinal fluid (CSF) volume also varies, with CSF volume showing decrease, increase, or no change. In the study conducted by Verges et al. (2016), the MRI scanning were performed in lowland before and within 6 h after returning to lowland, and this acute hypoxia markedly induced the increase in WM volume (0.7 ± 0.4%, *p* = 0.005), which may squeeze ventricle, and result in CSF volume reduction. In contrast, in the study of Kottke et al. (2015), a single sojourn to extreme altitudes was not associated with development of GM atrophy but lead to a decrease in brain WM fraction (–0.18%, *p* = 0.012), which may cause the ventricles to expand. In the observation of Zhang et al. (2012), because the subjects gradually reached their destination in the process of up to 10 days and returned to the plain within 10 days, the brain gradually adapted to hypoxia and various cerebrovascular barriers damaged by hypoxia (Michalicova et al., 2017) may recover to normal during slow reoxygenation, and so, only WM remained with local slight cytotoxic edema.

### Brain structural network and genetic variants

In a recent study, the modulation of HA adaptive genetic variants (single nucleotide polymorphisms [SNPs]) of EGLN1, EPAS1, and PPARA on the organizational network of the brain was investigated in Tibetan natives in the Qinghai-Tibet Plateau. The study showed that the HA adaptive genetic variants regulated the topological organization of large-scale structural brain networks (Guo et al., 2020). These findings provide novel insights into the genetic substrate and dynamic evolution of HA adaptive phenotypes in native Tibetans.

### Iron deposition in the brain

Iron is the most abundant transition metal found in the human brain, and is highly compartmentalized in subcortical nuclei. Iron plays an important role in the physiological functions and development of human brains (McAllum et al., 2020). MRI studies in patients with HACE revealed multiple hemosiderin depositions in the brain, predominantly in the corpus callosum (Kallenberg et al., 2008). In a recent study, brain iron levels were found to increase in the basal ganglions after 30 days of exposure to HA, and even remained slightly elevated 1 year after people return to sea level (Chen L. et al., 2017).

## Brain function after high altitude exposure

Detailed information is shown in Table 2.

### Electrophysiological characteristics

Event-related potentials (ERPs), obtained by time-locked averaging EEG, have been used to evaluate the electrophysiological processing of cognitive activity in various HA residents. Most studies of ERP use task-activated electrode points in the relevant brain regions for analysis, called regions of interest analysis which can control class I errors in statistical testing and better discernment of the effects of high altitude exposure on specific brain functions. Given the high temporal resolution, ERPs have been used to record the precise temporal sequence of brain processes. The onset of stimulation is shown to evoke a characteristic negative potential (N1) of ERP with a peak latency around 120 ms and is used as index of the attentional allocation of the early visual processing (Wang et al., 2014). Negative potential (N2pc: N2-posterior-contralateral) is an electrophysiological mark that embodies the visuospatial attention focusing on a target stimulus that supports visual search, which occurs approximately 280 ms after the presentation of a stimulus (Luck and Hillyard, 1994). The negative potential (N2) occurs approximately 200 ms after the presentation of a stimulus reflected the processing of conflict detection (Rueda et al., 2004). The positive potential (P50) occurs approximately 50 ms after the presentation of a stimulus and reflects a predominantly pre-attentional inhibitory filter mechanism that was admitted to the early sensory gating (Lijffijt et al., 2009). The positive potential (P2) is related to top−down processing, matching sensory input information with memory representations, which occurs approximately 200 ms after the presentation of a stimulus (Luck and Hillyard, 1994). The positive potential (P300/P3) of ERP occurs approximately 300 ms after the presentation of a stimulus, requiring detection, counting, or cognitive processing by the participants. The P3 component has generally been considered a late stage of information processing. Moreover, the time-frequency signal processing approach has been widely employed for the study of the energy distribution of ERP data across time and frequency. In particular, the time-frequency amplitude has been used extensively to investigate alpha, beta, delta, and theta powers with regard to ERP data recorded during cognitive tasks.

Earlier EEG studies mainly tested the electrophysiological processing of visual stimuluses after short-term exposure to HA. Two previous studies have observed EEG patterns during exposure to HA-induced hypoxia over time. One study found that the occipital alpha component was 25.5% at sea level, which changed at 3,500 m to 45.7, 15.8, 28.0, 30.3, and 33.2% on days 2, 7, 14, 21, and 28, respectively. The average amplitude was 17.3 μV at sea level, which changed at 3,500 m to 23.3, 11.8, 16.2, 17.3, and 19.8 μV on days 2, 7, 14, 21, and 28, respectively. These results indicate a cortical depression in the initial phase of HA exposure, followed by a gradual build-up of EEG waves during acclimatization (Selvamurthy et al., 1978). Another study was conducted in the lowland soldiers 7 days before ascending to altitude, and on the 7th and 30th days at 3,800 m, which found that acute acclimatization only decreased theta power, while chronic acclimatization discriminately increased alpha and beta powers but decreased delta power (Zhao et al., 2016). This study also found decreased alpha power and increased beta power within 7 days of returning to lowlands, which indicated a sustained higher level of cortical excitation during reoxygenation. After 5 days of HA exposure EEG frequency was increased (Forster et al., 1975). This is the only study that attempted to clarify neuronal activity during the “HA deadaptation reaction” (Zhou et al., 2012).

Recently, serial task-induced ERPs were measured in immigrants who had lived at Lhasa (3560 m) for more than 2 years. The HA immigrants showed reduced attentional resources with smaller P3 amplitudes (Wang et al., 2014; Qiu et al., 2021), reduced attention reactions in visual search tasks with lower N2pc amplitudes (Zhang et al., 2018a), overactive performance monitoring with larger error-related negative and correct-related negative amplitudes (Ma et al., 2015a), impaired response inhibition in the conflict-monitoring stage (Ma et al., 2015b; Wang et al., 2021) and visual executive ability (Ma et al., 2015c) with smaller P3 amplitudes, slowed stimulus-driven behaviors and P3 magnitudes of resource allocation (Ma et al., 2018a), impaired spatial manipulation ability with larger rotation-related negativity amplitudes (Ma et al., 2018b), decreased P50 mean amplitude and delay activity amplitude of mental rotation (Li et al., 2021), impaired spatial working memory with lower P2 and impaired verbal and spatial working memory maintenance with late-positive potentials (Ma et al., 2019b), and decreased alpha event-related desynchronization at the parietal-occipital regions and beta event-related desynchronization at the central-parietal regions within the time window (400–700 ms) in the mental rotation task (Xiang et al., 2021). Taken together, these electrophysiological studies showed that prolonged exposure to HA mainly impairs the late processing stage of cognition due to insufficient attention resources.

Rare electrophysiological studies of brain function were conducted on HA natives. In Bolivian children living at 3,700 m, reductions in the amplitude of delta and beta frequencies were recorded (Richardson et al., 2011). In Tibetan natives, an overactive executive ability with larger P3 and an impaired orienting ability with larger N1 were recorded only at extreme altitudes above 4,200 m, while below this threshold no cognitive impairments were observed, suggesting the existence of a threshold for the influence of HA exposure on brain function (Zhang et al., 2018b). The N2 difference wave was smaller in the 4,500 m group than in the groups living below 4,000 m, which indicated that the altitude threshold for impairment of cognition may be 4,000 m (Ma et al., 2019a).

### Brain neuronal activity

Given its high spatial resolution, functional magnetic resonance imaging (fMRI) is used to measure changes in blood dynamics caused by neuronal activity. In fMRI, the blood oxygenation level dependent (BOLD) effect is employed to identify and delineate neuronal activity. An initial fMRI study was conducted on individuals who stayed at Chacaltaya (5,260 m) for 5 weeks, and showed that the average magnitude of BOLD responses induced by visual stimulation was reduced in the occipital gyrus (Rostrup et al., 2005). Subsequently, serial fMRI studies were conducted on Han immigrant descendants who were born and raised in the Qinghai-Tibet Plateau for at least 17 years and later relocated to sea level for more than 1 year. These serial scans revealed the brain mechanisms underlying spatial working memory (Yan et al., 2011a), verbal working memory (Yan et al., 2011c), and food cravings (Yan et al., 2011d). Recently, a task fMRI study was conducted on military aviation personnel who have chronic intermittent exposure to HA, and found significant activation in the right middle frontal gyrus by a spatial working memory task (Tower of London paradigm) (Nisha et al., 2020). In soldiers who had garrisoned the frontiers in the Qinghai-Tibet Plateau for 2 years, the regional homogeneity (ReHo) of neuronal activity (Chen et al., 2016a), voxel-mirrored homo-topic connectivity in the bilateral visual cortex (Chen et al., 2016b), and amplitude of low-frequency fluctuations (ALFF) of resting-state neuronal activity (Zhang et al., 2017) were investigated. In the sea level college students who had immigrated to a plateau for 1 and 2 years, ReHo of neuronal activity was also studied (Chen X. et al., 2017; Wang et al., 2017), and further study on these students suggested that ReHo of neuronal activity in the bilateral putamen and bilateral pallidum can predict psychomotor impairment due to HA exposure (Chen et al., 2019b).

### Brain functional network

One study showed decreases of neuronal co-activation within the left/right frontoparietal network, sensorimotor network, and auditory network at resting state in lowland students after they had their college study at Lhasa for 2 years (Chen et al., 2021). Another study revealed changes of topological property of functional network of some important regions, showing alterations of degree centrality and nodal efficiency within the network after HA exposure for 2 years (Xin et al., 2020). The brain functional network alteration in the resting state may be the functional basic of executive control impairment.

## Conclusions of the findings in brain structures and functions

**FIGURE 2:**
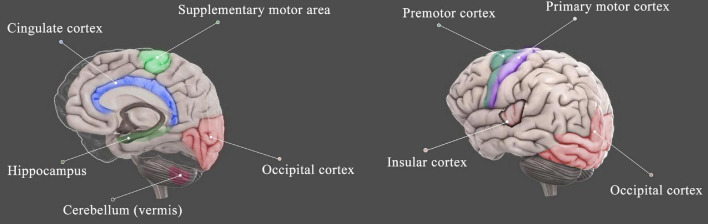
Schematic diagram shows the brain regions that were affected by HA exposure. The regions include the insular cortex (Zhang et al., 2010, 2013a, 2017; Yan et al., 2011b,d; Fan et al., 2016; Wei et al., 2017; Xin et al., 2020), occipital cortex (Zhang et al., 2010, 2013a, 2017; Yan et al., 2011a,b,c,d; Fan et al., 2016; Zhao et al., 2016; Wang et al., 2017, 2018; Wei et al., 2017; Xin et al., 2020; Xiang et al., 2021), cingulate cortex (Zhang et al., 2010, 2013b; Yan et al., 2011b; Fan et al., 2016; Verges et al., 2016; Chen X. et al., 2017; Wei et al., 2017; Wang et al., 2018), motor cortex (Zhang et al., 2010, 2013a; Chen et al., 2016a; Fan et al., 2016), cerebellum (Yan et al., 2011a; Zhang et al., 2012, 2013a,b; Xin et al., 2020), and hippocampus (Zhang et al., 2013b; Foster et al., 2015; Fan et al., 2016; Chen X. et al., 2017; Xin et al., 2020).

**FIGURE 3:**
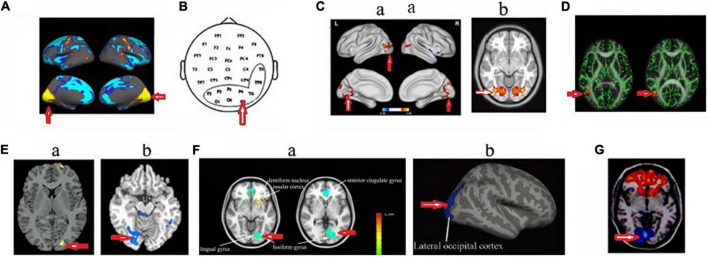
Changed structure and function in visual cortex in HA population. **(A)** Increased cortical thickness in sea-level college students who had a 30-day teaching at HA (Fan et al., 2016); **(B)** increased beta power in soldiers who had garrisoned at HA for 1 month (Zhao et al., 2016); **(C)** increased amplitude of low-frequency fluctuations (Zhang et al., 2017) **(a)** and voxel-mirrored homotopic connectivity (Chen et al., 2016b) **(b)** in soldiers who had garrisoned the frontiers at HA for 2 years; **(D)** The increased fractional anisotropy in Tibetan adolescents descending to sea level for 4 years (Zhang et al., 2013a); **(E)** Decreased gray matter volume (Zhang et al., 2010) **(a)** and decreased cerebrovascular reactivity (Yan et al., 2011b) **(b)** in the descendants of Han population who have immigrated to HA for several generations; **(F)** decreased cerebral blood flow (Wang et al., 2018) **(a)** and cortical thickness (Wei et al., 2017) **(b)** in HA Tibetan natives; **(G)** decreased the amplitude of low-frequency fluctuations in college students who studied at HA for 1 year (Wang et al., 2017). The arrow indicates the visual cortex.

**FIGURE 4:**
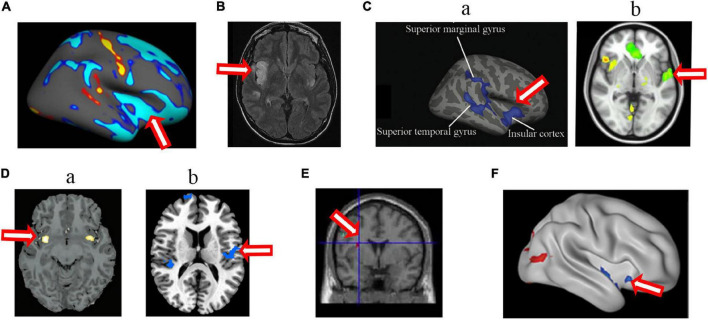
Changed structure and function in insular cortex in HA population. **(A)** The decreased cortical thickness in sea-level college students who had a 30-day teaching at HA (Fan et al., 2016); **(B)** hyperintense signaling in a woman after a rapid ascent to mountain (D’Arrigo et al., 2019); **(C)** the decreased sulcus depth (Wei et al., 2017) **(a)** and decreased cerebral blood flow (Wang et al., 2018) **(b)** in HA Tibetan natives; **(D)** the decreased gray matter volume (Zhang et al., 2010) **(a)** and longer dwelay of hemodynamic response (Yan et al., 2011b) **(b)** in the descendants of Han population who have immigrated to HA for several generations; **(E)** the increased gray matter volume in Tibetan adolescents descending to sea level for 4 years (Zhang et al., 2013a); **(F)** the decreased ALFF in soldiers who had garrisoned the frontiers at HA for 2 years (Zhang et al., 2017). The arrow indicates the insular cortex.

The regions of the brain found to be altered in the HA population included the bilateral insular cortex, occipital cortex, cingulate cortex, precentral gyrus, and hippocampus as well as cerebellum (summarized in Figure 2). Although multiple regions of the brain are affected during HA exposure, the most consistent findings involved the occipital visual cortex, covering lingual gyrus, fusiform gyrus, and precuneus (Figure 3), and insular cortex, including anterior insula and posterior insula (Figure 4). According to the review by Evans (2010), most of these regions are the key components of the cortico-limbic modulation of respiratory control and sensation. Supporting this review, GM structure and function in these regions showed correlations with pulmonary functions in several studies done on the HA population. For example, GM volume in the insular cortex correlated with vital capacity (Zhang et al., 2013a); BOLD signals in the insular cortex, thalamus, cerebellum, and precentral cortex correlated with inspiratory and expiratory reserve volumes (Yan et al., 2011b); the strength of functional connectivity in the right insular cortex to the right superior temporal gyrus showed significant positive correlation with the predicted forced expiratory volume in one second (FEV1) (Zhang et al., 2017); and GM volumes in the hippocampal and middle frontal gyri were significantly negatively correlated with vital capacity (Zhang et al., 2013a).

**FIGURE 5:**
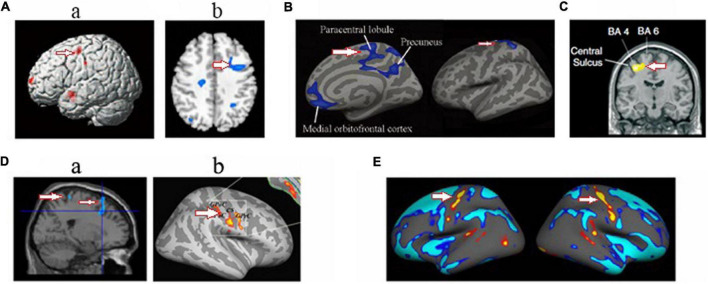
Changed structure and function in motor cortex in HA population. **(A)** The decreased gray matter volume (Zhang et al., 2010) **(a)** and cerebrovascular reactivity (Yan et al., 2011b) **(b)** in the descendants of Han population who have immigrated to HA for several generations; **(B)** the decreased cortical thickness in HA Tibetan natives (Wei et al., 2017); **(C)** the decreased white matter fiber volume projecting from motor cortex in Everest climbers (Di Paola et al., 2008). **(D)** the decreased the gray matter volume (Zhang et al., 2013b) **(a)** and increased regional homogeneity in soldiers who had garrisoned the frontiers at HA for 2 years (Chen et al., 2016a) **(b)**; **(E)** the increased cortical thickness in sea-level college students who had a 30-day teaching at HA (Fan et al., 2016). The arrow indicates the motor cortex.

In addition, structural and functional changes in the motor cortex, including Brodmann area 4, Brodmann area 6, and supplementary motor area (Figure 5), and cerebellum (mainly in vermis) were found in HA natives as well as HA immigrants. The cerebellum has diverse connections to the cerebrum, brain stem, and spinal cord, takes part in motor control, and is associated mainly with motor skills, visual-motor coordination, and balance (Swinny et al., 2005). Therefore, the abnormal structures of the motor cortex and cerebellum suggest deficits in motor coordination in HA population; however, there are no behavior tests so far to support these brain findings.

The hippocampus is thought to be a core brain region for spatial memory (Bellmund et al., 2018). Evidence from animal studies have showed that apoptotic death in the hippocampus may be involved in HA-induced memory impairments (Maiti et al., 2008), while the increased p-CREB, long-term potentiation, and synapses of the hippocampus were related to enhanced spatial learning and memory in mice exposed to intermittent hypobaric hypoxia (Zhang et al., 2005). Unexpectedly, however, only two MRI studies revealed impairments in the hippocampi of individuals who were exposed to HA for 2 years (Zhang et al., 2013b; Chen X. et al., 2017), which does not account for the changes in spatial cognition detected in various HA individuals. We suggest that the impaired visual cortex may be responsible for deficits in spatial cognition.

Many factors may contribute to the structural and functional changes of the visual cortex. The brain and retina have the highest oxygen uptake per unit mass of any body system (Parker and West, 1973; Country, 2017). Existing data show that vessel engorgement and tortuosity, optic disc hyperemia, and hemorrhages are often seen in Tibetans (Morris et al., 2006). Epidemiological data show that the prevalence of blindness in HA Tibetans was higher in Lhasa Tibetans than in lowland populations in the 50–59 years age range (Wang et al., 2013). The affected retinal vasculature decreases the blood supply to visual photoreceptor cells, reduces the activity in these cells, and thus decreases the excitement to visual cortical cells. The optic nerve axons are also thought to be injured by hypoxia (Chan et al., 2015). More importantly, the visual cortex is always directly affected by hypoxia, as during hypoxic events, neuronal activity and metabolism in the visual cortex are reduced (Rostrup et al., 2005; Vestergaard et al., 2016). Changes in the visual cortex may also be an outcome of increased exposure to ultraviolet (UV) radiation at HA (Wei et al., 2017). UV light has been shown to alter neuronal activity in the cortical structures involved in visual processing (Amir and Robinson, 1996), and environmental UV rays can induce apoptosis in the retinal and laminar ganglions within the visual system and brain (de Oliveira Miguel et al., 2002; Hollmann et al., 2016).

Altered neuronal activity in the insular cortex likely contributed to the regulation of ventilatory and cardiovascular functions while in the HA environment. As mentioned above, the relationship between the insular cortex and respiratory function has been demonstrated in HA populations. The insular cortex is connected with the hypothalamus, midbrain, pontine, and medulla oblongata (Gaytan and Pasaro, 1998; Bowman et al., 2013), as well as the diaphragm (Lois et al., 2009), all of which are involved in respiratory and cardiovascular sensation and control. Electrophysiological recording showed neural activity in the right anterior insular cortex mediated resting spontaneous breathing (Evans et al., 2009), while stimulation of the anterior insular cortex produced a set of specific alterations in the respiratory pattern (Aleksandrov et al., 2007). The injection of l-glutamate into the insular cortex induced apnea in rats (Cui et al., 2012). Numerous fMRI studies have shown that the anterior insular cortex plays an important role in air hunger (Evans, 2010; Parshall et al., 2012; Binks et al., 2014). The left insular cortex principally regulates parasympathetic activity and the right regulates sympathetic nerve activity. A structural impairment in the anterior insular cortex (Zhang et al., 2010; Wei et al., 2017) may be one reason why HA natives had a blunted hypoxic ventilatory response (West, 2002). The findings regarding the insular cortex support the hypothesis that the anterior insular and cingulate cortices are needed to process perturbation of the homoeostatic balance in extreme environments (Paulus et al., 2009; Zhang et al., 2010).

Nowadays, a large number of sea−level people migrate to high altitudes to travel or to work (Tianyi and Kayser, 2006). A review of the effects of altitude on the brain helps these people overcome the fear of high altitude. In addition, hypoxia is a very common disease in clinic. High altitude hypoxia is a natural model, and its damaged brain regions provide a theoretical basis for the therapeutic targets of clinical hypoxia diseases.

## Conclusion

The microstructural and functional alterations to the human brain in response to acute HA-induced stress or chronic acclimatization to HA environments have been studied *in vivo* mainly in the past 10 years. Among various HA populations, the most consistent findings involve changes in the visual, motor and insular cortices, and it has been found that the altered insular cortex may be related to cardiovascular or respiratory regulation.

At present, there are relatively few studies on HA brain, and thus the reliability of the results among these studies needs to be further confirmed. Some subjective or objective factors as follows may affect the results. (1) In MRI experiments, data acquisition and analysis are required to be carried out by different researchers; however, due to the limited conditions in the actual research (these data collection are difficult), some studies may not be double-blind. However, seeing that these results are relatively consistent, we suggest that the factor of human expectation is small. Insular cortex is the visceral sensory center, which can be expected to be affected by hypoxia, but the changes in the visual and motor cortices cannot be expected. Generally speaking, we think the results are relatively reliable. (2) VBM involves a voxel-wise comparison of the local concentration of GM between groups. Corrections for multiple comparisons are needed to avoid statistical class I errors. The results showed in some researches, however, cannot stand correction. With or without correction, similar HA exposure showed different results in different studies. For example, to study the effect of 2-year HA exposure on human brain, Zhang et al. (2013b) showed GM volume changes in multiple brain regions without statistical correction, while Chen X. et al. (2017) showed only one differential brain region under statistical correction. Of course, the subjects in Zhang et al. (2013b) are soldiers, and thus the physical training may partially aggravate the effect of hypoxia. (3) The procedure of VBM involves spatially normalizing high-resolution images from all the subjects in the study into the same stereotactic space, segmenting GM from the spatially normalized images and smoothing GM segments. Voxel-wise parametric statistical tests are performed by comparing the smoothed GM images from the two groups. All these steps involved in VBM could produce non-uniformity artifact. (4) Some studies reported no change in total brain volume, but only local changes. One way to think about it is that local brain changes account for only a small part of brain volume, and are not enough to affect the total brain. (5) Some longitudinal studies were conducted before and after HA exposure, in which subjects were scanned and rescanned at an interval, and thus MRI instrument-related factors may affect the morphological measurements. (6) At present, there are few high-intensity MRI machines on the HA areas. Previously, we have repeatedly performed MRI scans in Lhasa and found this phenomenon. (7) There were no lowland control group in some studies. In fact, in addition to the influence of plateau environment, subjects will be challenged by the changes of diet and cultural customs. In the future, when there will be more high-intensity MRI machines in the plateau area, and the machine performance cannot be disturbed by factors such as low pressure, it will be convenient to recruit a large number of HA population to carry out large sample research. At that time, the impact of HA environment on the brain will be more truly revealed.

There are still many questions that need to be answered, such as which mechanisms, neuronal, glial, and/or vascular, contribute to brain structure alterations? Exploring the genetic basis of brain structures in HA natives helps to understand the mechanisms of HA adaptation in the native population. The difference that exists between individual brains in response to HA is also an interesting issue. How about brain readaptation to lowlands after HA immigrants return to lowlands?

According to literature, there are several strategies that may be employed to protect brain injury in HA environment. (1) Cerebral edema contributes much to the severity of the ultimate brain damage. Gradual ascents are proven to be the best strategy for preventing HA cerebral edema (Houston and Dickinson, 1975; Basnyat and Murdoch, 2003; Joyce et al., 2018). Moreover, cerebral edema can be eliminated when HA immigrants leave a lower oxygen environment, such as descending to lowlands, being supplied with oxygen (Pines, 1978; Hackett and Roach, 2001; West et al., 2004; Davis and Hackett, 2017; Williamson et al., 2018), or inhaling a higher concentration of oxygen (West, 2003). (2) In the recent studies, salidroside has been illuminated as a promising neuroprotectant for preventing and treating ischemic stroke (see the review by Fan et al., 2020). Therefore, with higher efficacy and better safety profiles, salidroside can be used for preventing acute cerebral edema. (3) Reviews showed that the beneficial effects of living at HA on the cardiovascular and cerebrovascular systems, morbidity, and mortality are influenced not only by the level of altitude but also by lifestyle behaviors like physical activity, nutrition, smoking, and alcohol consumption (Burtscher, 2014; Mallet et al., 2021). Keep this in mind, we believe that forming a good lifestyle, such as no smoking and no drinking and proper exercise, will help to reduce brain damage. (4) Dietary restriction has been proved to increase the length of life in several species. Moderate dietary restriction, but not extreme dietary restriction, can reduce neurovascular damage after hypoxic-ischemia and confer long-term protection in neonatal brain (Tu et al., 2012) and thus dietary restriction may be an alternative protective strategy for brain hypoxic injury.

## Author contributions

XZ: investigation and writing—original draft. JZ: conceptualization, writing—review and editing, and funding acquisition. Both authors contributed to the article and approved the submitted version.
